# Case Report: lipoblastoma-like tumor of the tongue

**DOI:** 10.3389/fonc.2026.1941099

**Published:** 2026-09-29

**Authors:** Li Yi, Haiyan Yang, Wenxin Wu

**Affiliations:** Department of Pathology, The Second Hospital of Hebei Medical University, Shijiazhuang, China

**Keywords:** case report, clinicopathological features, head and neck tumor, lipoblastoma-like tumor, tongue

## Abstract

Lipoblastoma-like tumor (LLT) is a distinctive adipocytic neoplasm most commonly arising in the vulva of women. Lingual involvement is extremely rare. Herein, we present a case of LLT occurring in the tongue and review the relevant literature. The patient was a 41-year-old female who presented with a mass on the right lateral border of the tongue for 6 months. Microscopic examination revealed the typical features of LLT. Immunohistochemically, the tumor was positive for CD34 and vimentin, with partial positivity for CDK4 and S-100, and negative for Rb, p16, and MDM2. Fluorescence *in situ* hybridization (FISH) demonstrated no DDIT3 rearrangement or MDM2 amplification. Next-generation sequencing (NGS) detected no significant genetic alterations. The tumor was completely resected, and no recurrence was observed at 19 months of follow-up. Given the rarity of lingual LLT—of which only three cases, including ours, have been reported—this report expands the current knowledge base and emphasizes the need for accurate diagnosis and long-term patient surveillance.

## Introduction

Lipoblastoma-like tumors are a rare and emerging neoplastic entity of lipogenic origin, and were originally considered to primarily affect the vulvovaginal area in young patients (1, 2). However, with an increasing number of cases being identified, anatomical sites of LLTs have been found to include the inguinal region, mons pubis, scrotum, spermatic cord, limbs, paratesticular region, and pelvis (3–7). Involvement of the tongue is extremely rare, with only two sporadic cases documented to date (8, 9). One case report describes a unique adipocytic tumor of the tongue that presents the characteristic findings of LLT but exhibits a mosaic Rb pattern and a hemizygous loss of the RB1 locus. The other tongue-involved case harbors an IDH1 mutation, a hitherto undescribed molecular alteration (10). Here, we describe an additional case of lingual LLT that shares similar morphological and immunohistochemical features with prior reports, while showing no DDIT3 or MDM2 gene rearrangements or amplifications, and negative findings on comprehensive NGS.

## Case presentation

A 41-year-old woman visited our hospital with a 6-month history of an incidental finding of a mass at the right lingual margin. The swelling grew slowly without discomfort such as pain, numbness or itching. No additional auxiliary examinations were performed prior to surgery, and the patient underwent complete local excision of the tumor.

Macroscopically, the tumor was described as an ash-yellow soft mass with dimensions of 1×1×0.8cm. Histopathological examination showed a partially well-demarcated tumor beneath the mucosa (**Figure 1A**), with focal infiltration into adjacent striated muscle (**Figure 1B**). Notably, the tumor exhibited a lobulated morphology, with delicate fibrous septa separating the lobules (**Figure 1C**). Additionally, the myxoid stroma contained bland spindle cells without any atypical features (**Figure 1D**). The tumor was predominantly composed of spindle cells and mature adipocytes, and some cells exhibited lipoblast-like morphology (**Figure 1E**). These lipoblast-like cells contained variably sized vacuoles, resembling uni-, bi-, or multi-vacuolated lipoblasts (**Figure 1F**).

**Figure 1:**
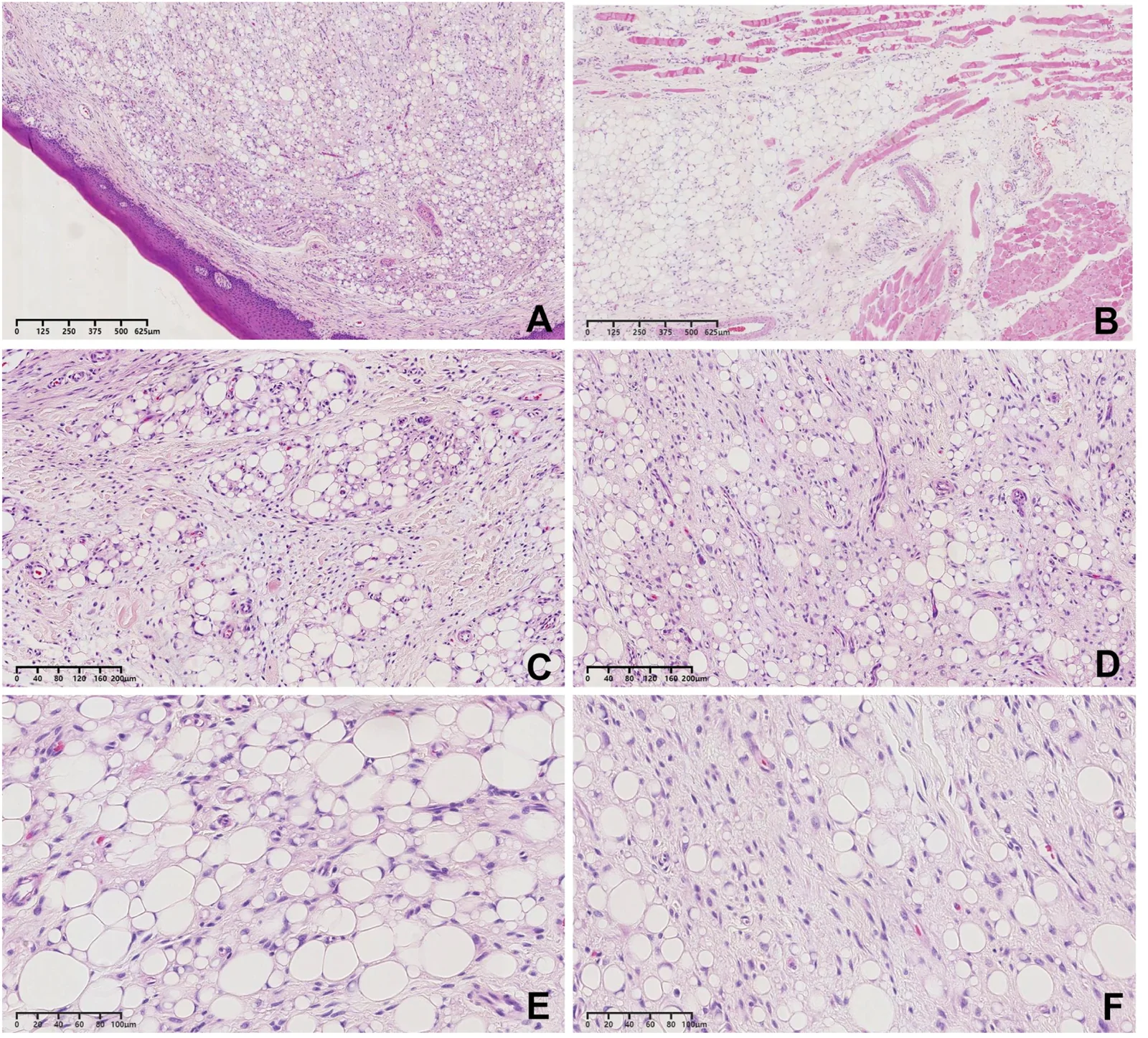
Histopathological features of the lingual lipoblastoma-like tumor. **(A)** The tumor is located beneath the squamous mucosa and is relatively well demarcated in most areas (H&E, ×4). **(B)** Focal infiltration of tumor cells into the surrounding striated muscle tissue is observed (H&E, ×4). **(C)** The tumor exhibits a characteristic lobulated architecture, with delicate fibrous septa interspersed between the lobules (H&E, ×20). **(D)** The myxoid stroma contains numerous small, thin-walled blood vessels (H&E, ×10). **(E, F)** The tumor is predominantly composed of bland spindle cells and mature adipocytes, admixed with lipoblast-like cells containing variably sized vacuoles (H&E, ×20).

By immunohistochemistry, all tumor cells including spindle cells, mature adipocytes, and lipoblast cells were positive for CD34 (**Figures 2A,B**) and vimentin, but negative for MDM2 and p16. Mature adipocytes and lipoblasts showed partial positivity for CDK4 and S-100 (**Figure 2C**), and Rb protein expression was absent (**Figure 2D**). Additionally, the Ki67 labeling index (**Figure 2E**) indemonstrated extremely low proliferative activity.

**Figure 2:**
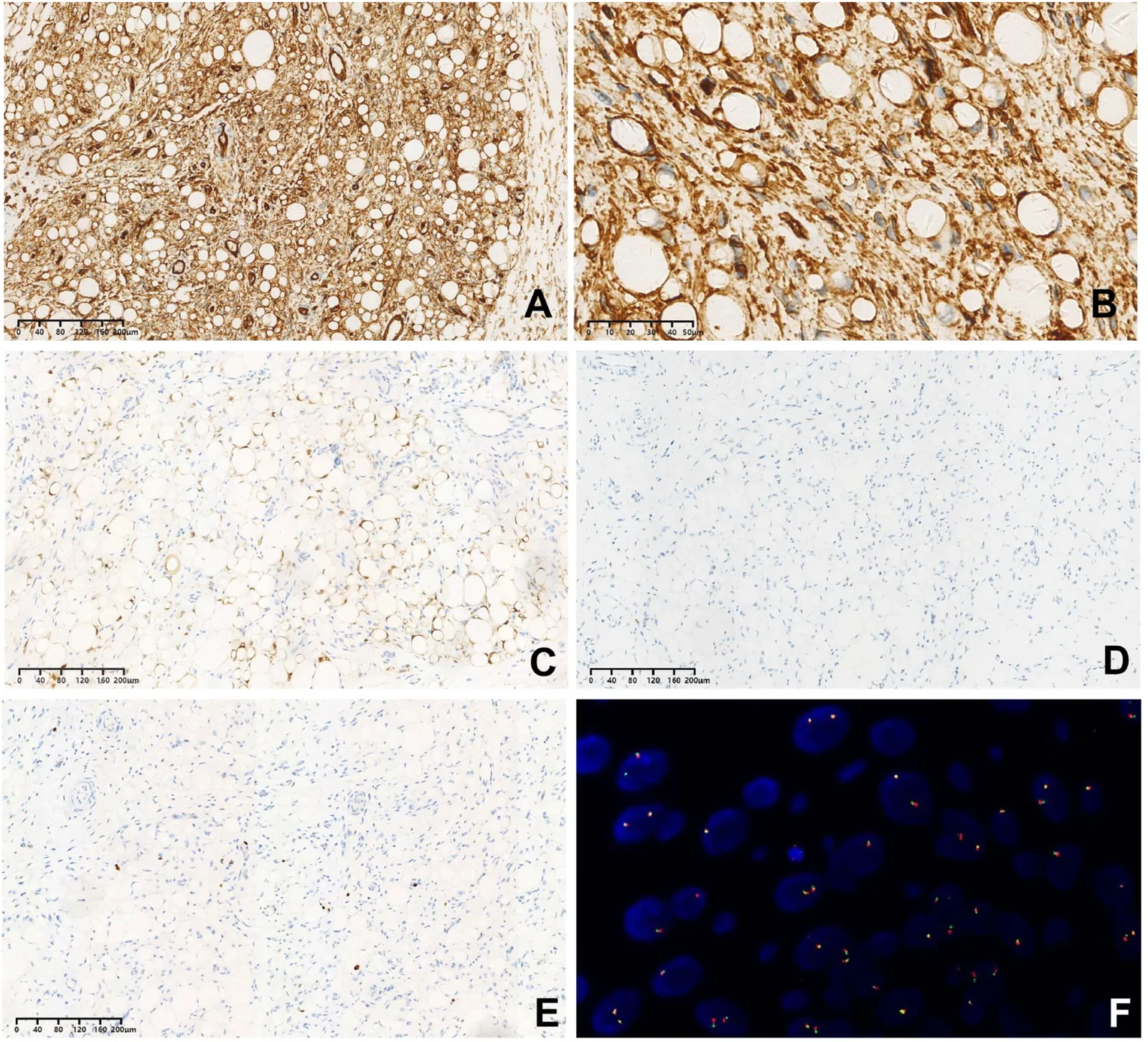
Immunohistochemical and molecular features of the lingual lipoblastoma-like tumor. **(A)** Diffuse cytoplasmic positivity for CD34 is observed in tumor cells (IHC, ×10). **(B)** At higher magnification, CD34 staining is detected in both spindle cells and lipoblast-like cells (IHC, ×40). **(C)** Lipoblast-like cells show partial cytoplasmic positivity for S-100 (IHC, ×10). **(D)** Complete loss of Rb nuclear expression is evident in tumor cells (IHC, ×10). **(E)** The Ki-67 labeling index is extremely low, with only scattered positive nuclei (IHC, ×10). **(F)** Interphase FISH analysis demonstrates the absence of DDIT3 gene rearrangement, as indicated by the lack of split signals (FISH, ×100).

The FISH assay results indicated the absence of DDIT3 rearrangements (**Figure 2F**), as well as no amplification of MDM2. To further investigate potential molecular drivers, high-throughput NGS was performed on tumor tissue. High-throughput NGS was performed using the Human 188-Gene Mutation Detection Kit (Jiyinjia, Suzhou, China) on the Gene+Seq-2000 platform. This targeted panel covers 188 cancer-related genes, including full coding regions of 27 genes, intronic/fusion regions of 17 genes, and partial coding regions of 156 additional genes. Key genes relevant to this case—PIK3CA, MTOR, TSC1, TSC2, CDKN2A, CDKN2B, RB1, IDH1, IDH2, TP53, MDM2, and MMR genes—are all covered. Sequencing achieved an average depth of 4,191× with 100% of target bases covered at >100× and a Q30 of 91%. The assay returned negative results for all tested alterations.

## Diagnostic assessment, therapeutic intervention, follow-up, and outcomes

The diagnostic workup included histopathological examination, immunohistochemistry, FISH, and NGS. The tumor was confirmed as LLT based on characteristic morphological features (lobulated architecture, myxoid stroma, bland spindle cells, and lipoblast-like cells) and immunophenotype (CD34+, Rb−, MDM2−, p16−, partial S-100+ and CDK4+), combined with the absence of DDIT3 rearrangement and MDM2 amplification. Complete surgical resection was performed with negative margins. No adjuvant therapy was administered. At 19 months post-surgery, the patient remained disease-free with no evidence of clinical recurrence.

## Discussion

Lingual involvement of LLT remains exceptionally rare, with only two prior cases documented to date (8, 9). Our case represents the third reported instance of lingual LLT (**Table 1**), and its comparative analysis with these prior reports reveals both consistent features and notable molecular heterogeneity.

**Table 1:** Summary of all published cases of Lipoblastoma-Like Tumor of the tongue.

Case	Sex	Age	Size (cm)	Clinical presentation	Surgical Margins	Molecular genetic	Follow-up (month)
Li et al. (5)	Female	65	1.5 × 1.7 × 4.7	Incidental finding of a multinodular mass at the right lingual margin for 1 month	Negative (frozen margin)	IDH1 (R132C) mutation; no DDIT3, PLAG1, MDM2, CDK4 rearrangements/amplification; no RB1 gene deletion	10 (no recurrence)
Yoshihiko et al. (6)	Female	62	0.5 × 0.55	Slowly developing painless nodule on the dorsal tongue for 7 years	Not explicitly stated (likely negative, as no recurrence)	Hemizygous deletion of RB1 locus (in 36% of cells); no MDM2 amplification	60 (no recurrence)
Present case	Female	41	1 × 1 × 0.8	Incidental finding of a mass at the right lingual margin for 6 months	Negative	No significant alterations detected (including PIK3CA, MTOR, TSC1, CDKN2A/B, RB1, IDH1, TP53); MSS	19 (no recurrence)

From a clinical perspective, the presenting features of lingual LLT are nonspecific and overlap with those of more common tongue lesions. In our case, the patient presented with a slowly enlarging, painless mass at the right lateral lingual margin over 6 months, without ulceration, bleeding, or functional impairment. These indolent clinical features are shared by benign lingual tumors such as lipoma, fibroma, or granular cell tumor, but may also be seen in low-grade sarcomas, including myxoid liposarcoma (3). This diagnostic challenge is further highlighted by the existence of rare oral lipoma variants—such as those of the buccal fat pad (11)—which can mimic other entities and underscore the adjunctive role of imaging. In our case, although MRI was not obtained preoperatively, it would typically show a well-defined or partially infiltrative mass with signal intensities reflecting fatty and myxoid components, findings that contribute to but do not definitively establish the diagnosis (8). For clinicians, the key takeaway is that a painless, slowly growing lingual mass in an adult, particularly with imaging features suggesting a fatty or myxoid lesion, should raise the possibility of LLT as a rare differential, and warrant complete excision with histopathological and molecular confirmation.

Grossly, LLT typically presents with well-defined margins; however, in our case, the tumor showed focally indistinct borders and infiltration into surrounding muscle. Similarly, Li et al. (8) observed a small satellite nodule within adjacent muscle in their lingual case. Recurrence and metastasis have been reported in some LLT cases, with an overall recurrence rate of 23.81% (10/42), primarily attributed to positive surgical margins (2). This suggests that lingual LLT may be particularly prone to ill-defined borders, and achieving adequate surgical margins is critical for preventing recurrence. In all three reported lingual cases, including ours, surgical margins were negative and no recurrence was observed at last follow-up, consistent with the broader literature.

LLT lacks specific immunohistochemical markers (1–4, 12). In our case, we observed strong CD34 positivity, loss of Rb expression, negativity for MDM2 and p16, partial CDK4 and S-100 staining, and a low Ki-67 index—findings consistent with previous reports. The most consistently reported alteration is aberrant Rb protein expression, often presenting as a mosaic pattern of partial or complete loss in subsets of tumor cells. In Anderson et al. ‘s series (4), approximately half of the cases showed loss of Rb expression, with no significant site-related differences. Notably, all three lingual LLT cases—including ours—exhibited Rb protein loss, suggesting that this feature may be more prevalent in lingual tumors, although this observation may be influenced by the small sample size.

In the tongue LLT case reported by Sugita et al. (9), RB1 gene deletion was detected, and the morphological and immunohistochemical characteristics supported the diagnosis of LLT, they believe that is an unusual LLT with Rb loss. However, the RB1 gene deletion was not detected in the other two tongue LLT cases (Li et al.’s and ours). The discordance between negative NGS findings for RB1 and complete loss of Rb protein expression by IHC raises the possibility of non-genomic or epigenetic mechanisms. Potential explanations include RB1 promoter hypermethylation leading to transcriptional silencing, microRNA-mediated post-transcriptional regulation, or post-translational protein degradation. The mosaic pattern of Rb loss observed in our case—and in prior reports—suggests a hemizygous rather than biallelic alteration, which may not be detected by standard NGS. Further studies incorporating methylation-specific PCR or RB1 FISH would be valuable to clarify the underlying mechanisms. This discrepancy suggests that Rb protein loss in LLT may arise through mechanisms other than simple genomic deletion, potentially including epigenetic silencing or post-translational modifications, and that the genetic heterogeneity of LLT may be more pronounced than previously appreciated.

FISH also ruled out DDIT3 rearrangement and MDM2 amplification. These negative molecular findings are diagnostically valuable: they effectively exclude MLPS (by absence of DDIT3 rearrangement) and ALT/WDLPS (by absence of MDM2 amplification). Beyond liposarcomatous entities, the differential diagnosis of lingual LLT also includes several benign adipocytic neoplasms that may present in the oral cavity. Spindle cell lipoma, which commonly occurs in the head and neck region of adults, typically shows a mixture of bland spindle cells and mature adipocytes in a myxoid or collagenous stroma, with characteristic CD34 positivity. Moreover, the lack of RB1 mutation or homozygous deletion, combined with the mosaic Rb pattern, suggests a hemizygous rather than biallelic RB1 alteration, distinguishing LLT from spindle cell/pleomorphic lipoma. Classical lipoblastoma, a benign tumor of embryonic fat most frequently seen in children under 3 years, also exhibits lobulated architecture and immature adipocytes, but it is characterized by PLAG1 rearrangements—which are absent in LLT—and typically lacks the CD34+/Rb− immunophenotype. Thus, while clinical presentation may overlap, the combined assessment of histomorphology, immunohistochemistry, and targeted molecular testing enables reliable distinction among these entities.

To further explore the molecular landscape of our case, we performed a targeted NGS panel that included genes previously implicated in LLT or its differential diagnoses. This panel was designed with the following rationales: (1) PIK3CA and MTOR were included because activating mutations in these genes have been reported in a vulvar LLT case with widespread metastasis (4, 13), suggesting a potential link to aggressive behavior; (2) TSC1 and CDKN2A/CDKN2B/MTAP were tested given their reported deletions in a subset of LLTs, though their clinical significance remains uncertain (3); (3) IDH1 was specifically included because it was identified in the prior lingual LLT case reported by Li et al. (8), raising the possibility of a site-specific or recurrent alteration; and (4) MMR genes (MLH1, MSH2, MSH6, PMS2) and TP53 were added as part of a broader exploratory approach to assess whether any known sarcoma-associated pathways might be dysregulated, despite their lack of established relevance in LLT—we acknowledge this as a hypothesis-generating effort. In our case, NGS detected no alterations in any of these genes, and the tumor was microsatellite-stable (MSS). While these negative findings do not support a role for these pathways in this particular tumor, they contribute to the growing body of evidence that LLT lacks a unifying molecular driver and may instead represent a genetically heterogeneous group of neoplasms.

While the comprehensive negative molecular profile reinforces the diagnosis and argues against a high-grade sarcoma mimic, it cannot, in isolation, be interpreted as definitive proof of a favorable prognosis, given the rarity of this entity and the documented aggressive behavior in a subset of cases. We recommend that patients with lingual LLT undergo regular clinical follow-up at 6-month intervals for the first 2 years, and annually thereafter up to 5–10 years, to monitor for potential late recurrence. This follow-up schedule is particularly important given the documented recurrence rate in LLT cases with positive or indeterminate margins, and the focal infiltration observed in our case. This case also illustrates the value of a multi-platform molecular testing approach—integrating FISH and NGS—in diagnostically challenging or atypically presenting neoplasms.

### Strengths

A major strength of this report lies in the comprehensive molecular characterization of this rare tumor, incorporating both FISH and NGS analyses, which enabled the systematic exclusion of key differential diagnostic entities.

### Limitations

Several limitations should be acknowledged. First, the inherent nature of a single-case report restricts the generalizability of our observations. Second, the follow-up duration of 19 months is insufficient to definitively rule out late local recurrence—a possibility that cannot be excluded given the documented 24% recurrence rate in LLT cases overall and the focal infiltration into adjacent muscle observed in our case. Third, the finding of Rb protein loss without detectable RB1 genomic deletion may not be broadly generalizable, and further studies are needed to clarify the underlying mechanisms.

## Conclusion

In summary, we report a rare case of lingual LLT with no significant gene variants, expanding the documented anatomic spectrum of this tumor and enhancing our understanding of its molecular landscape. This report underscores the importance of accurate histopathological and molecular diagnosis, and reinforces the need for comprehensive clinical evaluation and long-term follow-up to optimize patient management and avoid unnecessary interventions.

## Data Availability

The original contributions presented in the study are included in the article/supplementary material. Further inquiries can be directed to the corresponding author.
